# Significant bleeding from Meckel’s diverticulum after blunt abdominal trauma: a case report

**DOI:** 10.1186/s13256-018-1799-4

**Published:** 2018-09-19

**Authors:** Sharfuddin Chowdhury, Abdullah Maher Alenazi, Yam Alwi Alharthi

**Affiliations:** 1 King Saud Medical City, 7790 Al-Imam Abdul Aziz Ibn Muhammad Ibn Saud, Ulaishah, Riyadh, 12746 Kingdom of Saudi Arabia; 2 King Saud University, Qurtobah, Riyadh, Kingdom of Saudi Arabia

**Keywords:** Meckel’s diverticulum, Wounds and injuries, Hemorrhage

## Abstract

**Background:**

Meckel’s diverticulum, with an incidence of 2%, is the most common congenital anomaly in the gastrointestinal tract. Its main complications are perforation, obstruction, and bleeding. A few studies have reported that blunt abdominal trauma may result in perforation or obstruction to Meckel’s diverticulum. However, reports of significant major intestinal bleeding from Meckel’s diverticulum as a complication of blunt abdominal trauma is rare. This paper present what we believe to be the first reported case of significant intestinal bleeding from a Meckel’s diverticulum following blunt abdominal trauma.

**Case presentation:**

A 12-year-old Saudi boy of Arab ethnicity presented to the King Saud Medical City emergency department with bleeding per rectum and mild abdominal pain following blunt trauma to his abdomen. On examination, his abdomen was slightly tender, bowel sounds were present, and he was hemodynamically stable. During admission, rectal bleeding was ongoing. On day 3 he deteriorated with decreasing blood pressure and hemoglobin, and increasing pulse rate with fever. After resuscitation and stabilization, he was urgently taken to the operating room for further diagnostic management and treatment. His nasogastric tube revealed bile without blood, and an intraoperative colonoscopy revealed altered blood within his whole colon and terminal ileum without a definite bleeding site. A laparotomy was performed, and an injured branch of the mesenteric artery supplying the Meckel’s diverticulum was identified as the source of the significant arterial bleeding. Suture ligation controlled the bleeding, and the Meckel’s diverticulum was resected. The patient remained stable after that until discharge without any further intestinal bleeding.

**Conclusion:**

Identifying bleeding as a complicated Meckel’s diverticulum following blunt trauma to the abdomen can be challenging due to its low incidence and difficulties while making the diagnosis.

## Background

The first description of Meckel’s diverticulum was by Fabricius Hildanus in the sixteenth century [1, 2]. Johann Meckel then described the embryologic and pathologic features of this anomaly in 1809 [3]. Meckel’s diverticulum, with an incidence of 2%, is the most common congenital anomaly in the gastrointestinal tract [4]. The vast majority of cases are asymptomatic. Its main complications are perforation, obstruction, and bleeding [5]. A few studies have report that blunt trauma to the abdomen may result in perforation or obstruction of Meckel’s diverticulum [1, 6–8]. However, significant major intestinal bleeding from Meckel’s diverticulum as a complication of blunt abdominal trauma seems to be rare and has hardly ever been reported. We present a case of significant intestinal bleeding from a Meckel’s diverticulum following blunt abdominal trauma.

## Case presentation

**Fig. 1:**
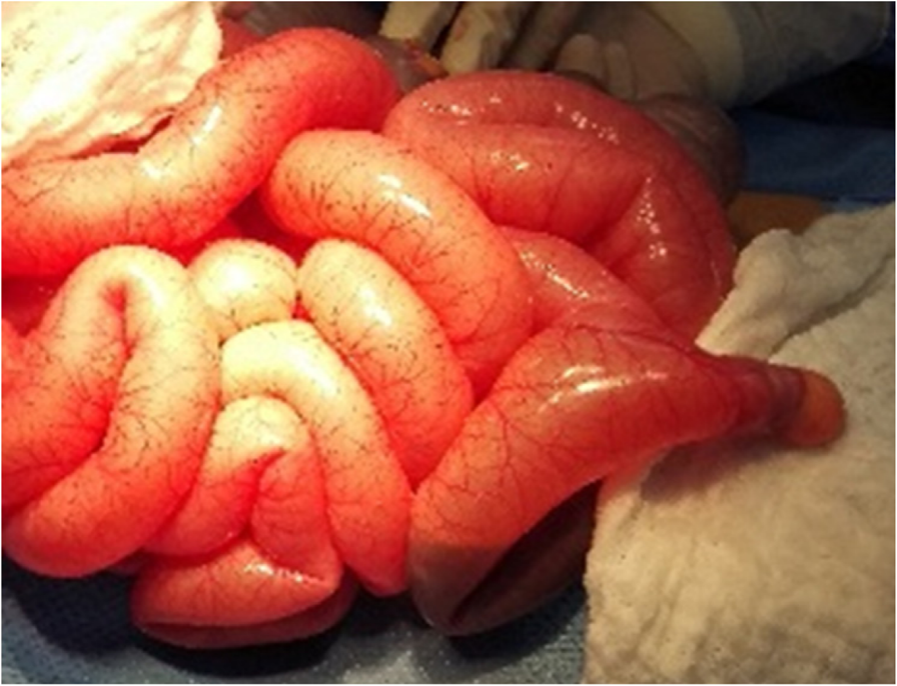
Bleeding Meckel’s diverticulum

**Fig. 2:**
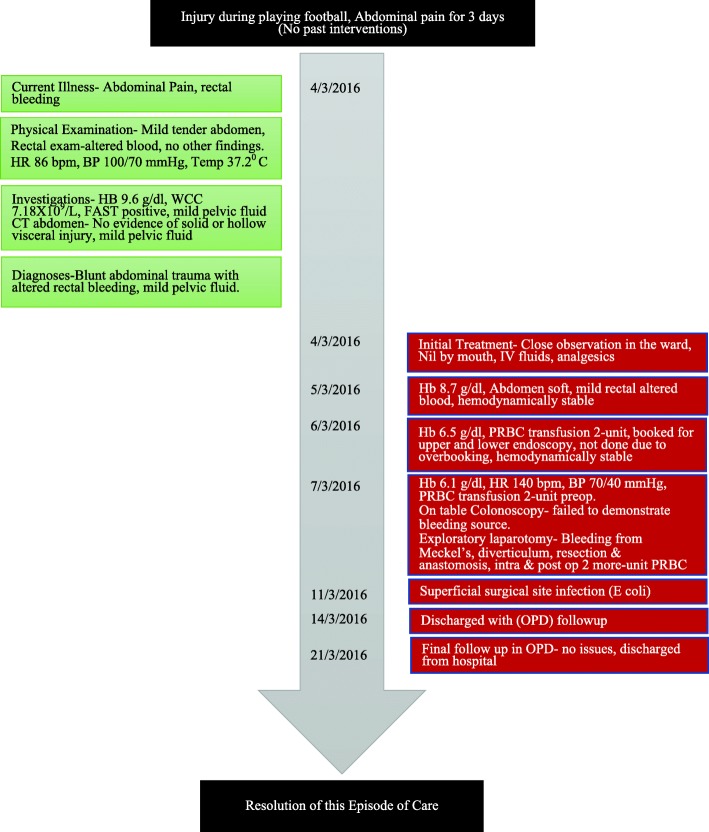
Timeline. *BP* blood pressure, *bpm* beats per minute, *CT* computed tomography, *FAST* focused assessment with sonography for trauma, *Hb* hemoglobin, *HR* heart rate, *IV* intravenous, *OPD* out-patient department, *PRBC* packed red blood cells, *WCC* white blood cell count

A 12-year-old Saudi boy of Arab ethnicity, with no past relevant medical, surgical, family, or psychosocial history, presented to the King Saud Medical City emergency department with bleeding per rectum and mild abdominal pain 3 days after blunt abdominal trauma while playing football. On examination, his abdomen was slightly tender and bowel sounds were present. Initially, focused assessment with sonography for trauma (FAST) showed mild intraperitoneal fluid in his pelvis. A subsequent computed tomography (CT) scan of his abdomen revealed mild pelvic hemoperitoneum, but there was no definite solid or hollow visceral injury. So, he was admitted to the general ward for serial abdominal observation. On admission to the surgical ward, he was stable with a heart rate (HR) of 86 beats per minute (bpm) and blood pressure (BP) of 100/70 mmHg. His hemoglobin (Hb) was 9.6 gm/dl. We kept him nil per mouth and a nasogastric tube was inserted. The intake and output chart was regularly calculated. Serial Hb monitoring showed a continuous fall in his Hb level during the following days. He continued to have altered rectal bleeding. On day 3 his Hb dropped to 6.1 gm/dl, despite having received 2 units of packed red blood cells (PRBC). He started to develop a mild fever with a temperature of 37.8 °C, tachycardia with a HR of 140 bpm, and the BP dropping to 70/40 mmHg. He was resuscitated and stabilized with intravenously administered crystalloid fluids and 2 units of PRBC. He did not require any inotropes or vasopressors. Due to this clinical deterioration, he was urgently taken to the operating room for further diagnostic and therapeutic management. The nasogastric tube revealed bile without hematemesis. After initiating general anesthesia, an on-table colonoscopy was performed to identify the bleeding site. There was altered blood within the whole colon and terminal ileum without a definite bleeding site. Therefore, we proceeded to a laparotomy. During the laparotomy, an injured Meckel’s diverticulum was identified as the source of the significant arterial bleeding (Fig. 1). Proximal to the diverticulum the small bowel was collapsed and did not show any blood. The bleeding from a branch of the mesenteric artery was stopped, and the Meckel’s diverticulum was resected. Histology revealed vascular injury due to trauma and gastric mucosa within the diverticulum. He required another 2 units of PRBC transfusion during and after the operation, and this increased his Hb to 10.2 gm/dl. He remained stable after that and developed postoperative superficial surgical site infection on day 4. He was discharged home on postoperative day 7 with instructions for wound care at a local clinic and our out-patient department (OPD) follow up after 1 week. No further complications were found during his OPD visit, and he was discharged from our hospital (Fig. 2, Timeline).

## Discussion

In a non-traumatic scenario, lower gastrointestinal bleeding is the most common complication of Meckel’s diverticulum; it ranges from 25 to 50% [9]. It is usually caused by the presence of either an ectopic gastric or pancreatic mucosa eroding the lumen of the diverticulum and its blood supply [10]. However, in this case, the blunt traumatic insult to our patient’s abdomen resulted in a tear in the mesenteric arteries supplying the Meckel’s diverticulum, leading to continuous intraluminal arterial bleeding. The melena was caused by the Hb in the blood being altered by digestive enzymes and intestinal bacteria. Abdominal bleeding caused the abdominal pain; the abdominal pain is usually due to distention and stretching of the bowel lumen in intraluminal bleeding, or due to irritation from the blood in the abdominal cavity in extraluminal bleeding. The absence of both criteria in this case made the bleeding painless. The mild pain on presentation of this patient can be explained by the impact of the trauma itself.

Preoperative diagnosis of Meckel’s diverticulum is very challenging and can be as low as 5.7% [11]. The introduction of a technetium-99m pertechnetate scan may help in correctly identifying Meckel’s diverticulum by being specific to its ectopic gastric mucosa. When in doubt, laparoscopy or laparotomy are the most definitive diagnostic modalities. Small bowel injury due to blunt abdominal trauma accounts for 1 to 7% of all intra-abdominal injuries in children. The most common injury is jejunum followed by ileum, duodenum, colon, and stomach injuries [12–14].

This low incidence along with the scarcity of Meckel’s diverticulum might be the reason behind the rarity of reports concerning both matters. To the best of our knowledge, the present report is the first to describe bleeding associated with Meckel’s diverticulum as a complication of blunt abdominal trauma. However, perforation and obstruction associated with Meckel’s diverticulum due to blunt abdominal trauma have been described by a few reports [1, 6–8]. Due to this low incidence, the suspicion of a bleeding Meckel’s diverticulum following blunt abdominal trauma is very low and rarely considered, which makes its diagnosis extremely difficult for surgeons and gastroenterologists. As an alternative treatment option, embolization of the bleeding mesenteric artery by interventional radiology could be considered. In this case, the deterioration of our patient’s hemodynamics, dropping Hb, and rising temperature, led us to opt for urgent surgical intervention as the more sensible option, and it proved to be successful.

## Conclusions

Bleeding Meckel’s diverticulum due to blunt abdominal trauma is a rare surgical condition. Identifying bleeding as a complicated Meckel’s diverticulum can be challenging due to its low incidence and difficulties while making the diagnosis. The surgeon should always consider this as a differential diagnosis in cases of rectal bleeding following abdominal injury.
